# RadioGuide-DCN: A Radiomics-Guided Decorrelated Network for Medical Image Classification

**DOI:** 10.3390/bioengineering13010046

**Published:** 2025-12-31

**Authors:** Lifeng Guo, Ying Fu, Shi Tan, Qi Wang, Yangan Zhang, Xiaohong Huang, Xueguang Yuan

**Affiliations:** 1 School of Electronic Engineering, Beijing University of Posts and Telecommunications, Beijing 100876, China; guolifeng@bupt.edu.cn (L.G.); wangqi@bupt.edu.cn (Q.W.); zhang@bupt.edu.cn (Y.Z.); 2 Department of Ultrasound, Peking University Third Hospital, Beijing 100191, China; yingfu312@gmail.com (Y.F.); tanshi@gmail.com (S.T.); 3 School of Computer Science, Beijing University of Posts and Telecommunications, Beijing 100876, China; huangxh@bupt.edu.cn

**Keywords:** medical image classification, radiomics, feature fusion

## Abstract

Medical imaging is an indispensable tool in clinical diagnosis and therapeutic decision-making, encompassing a wide range of modalities such as radiography, ultrasound, CT, and MRI. With the rapid advancement of deep learning technologies, significant progress has been made in medical image analysis. However, existing deep learning methods are often limited by dataset size, which can lead to overfitting, while traditional approaches relying on hand-crafted features lack specificity and fail to fully capture complex pathological information. To address these challenges, we propose RadioGuide-DCN, an innovative radiomics-guided decorrelated classification network. Our method integrates radiomics features as prior information into deep neural networks and employs a feature decorrelation loss mechanism combined with an anti-attention feature fusion module to effectively reduce feature redundancy and enhance the model’s capacity to capture both local details and global patterns. Specifically, we utilize a Kolmogorov–Arnold Network (KAN) classifier with learnable activation functions to further boost performance across various medical imaging datasets. Experimental results demonstrate that RadioGuide-DCN achieves an accuracy of 93.63% in BUSI image classification and consistently outperforms conventional radiomics and deep learning methods in multiple medical imaging classification tasks, significantly improving classification accuracy and AUC scores. Our study offers a novel paradigm for integrating deep learning with traditional imaging approaches and holds broad clinical application potential, particularly in tumor detection, image classification, and disease diagnosis.

## 1. Introduction

Medical imaging technologies—such as CT, MRI, X-ray, and ultrasound—are fundamental to modern clinical diagnosis, treatment planning, and efficacy evaluation. In this study, medical image classification refers to supervised disease-related categorization tasks based on commonly used clinical imaging modalities, including X-ray, ultrasound, and magnetic resonance imaging (MRI), such as disease presence identification and pathological type discrimination. As imaging techniques advance, the volume and complexity of imaging data have surged, presenting challenges in information extraction and fueling demand for automated, intelligent analysis. This data growth has driven the innovative application of artificial intelligence (AI), especially deep learning (DL), in precision medicine. High-dimensional imaging data enables training of complex models capable of discovering subtle pathological patterns beyond human visual perception.

Deep learning, particularly convolutional neural networks (CNNs), has transformed medical image analysis by automatically learning multi-level feature representations, significantly surpassing traditional methods reliant on handcrafted features [1]. CNNs extract both low-level textures and high-level semantics, enhancing tasks like classification, segmentation, and detection [2]. Landmark architectures—AlexNet, VGGNet, ResNet, DenseNet—have addressed issues such as vanishing gradients and feature reuse, achieving robust performance across various modalities (X-ray, CT, MRI, PET) and clinical applications (tumor detection, organ segmentation) [3]. Vision Transformers (ViTs), which leverage self-attention to model global dependencies, have further expanded model capability. Hybrid CNN-ViT models, integrating local and global feature extraction, offer even more comprehensive representations, addressing the multi-scale, distributed nature of diagnostic information in medical images.

Alongside DL, radiomics has become prominent. Radiomics extracts large numbers of quantitative features (shape, intensity, texture, wavelet) from medical images, enabling non-invasive assessment of tissue characteristics and pathology [4]. Radiomic features reveal tumor heterogeneity, disease progression, and treatment response, particularly valuable in oncology [5]. By converting images into high-dimensional data, radiomics facilitates predictive modeling and can be integrated with other omicsdata for personalized medicine. However, radiomics is constrained by complex, standardized workflows (e.g., image normalization, accurate ROI segmentation), and its performance is sensitive to feature selection and reproducibility [4].

To leverage both approaches, multimodal fusion—combining deep learning and radiomic features—has been widely studied [6]. DL features provide high-level, nonlinear representations, while radiomics offers interpretable, knowledge-driven descriptions. Effective fusion mechanisms (early, middle, late fusion) integrate complementary information, building more robust models. Additionally, attention mechanisms—inspired by human visual cognition—allow models to dynamically focus on task-relevant regions or features, improving classification and segmentation accuracy.

In summary, medical image classification is evolving from handcrafted-feature-based approaches, to deep learning-based automatic feature extraction, and now towards integrated, multimodal analysis using radiomics and attention mechanisms. The main challenge is how to fully utilize the complementary strengths of deep learning and radiomics under real-world constraints (limited data, high annotation cost), while achieving accuracy, robustness, and interpretability. This study is proposed to address these core issues. Different from prior radiomics-guided deep learning frameworks that primarily focus on feature concatenation or correlation suppression, this work explicitly addresses an overlooked issue: in heterogeneous radiomics-deep representations, discriminative complementary information often resides in low-correlation feature dimensions, which can be suppressed by conventional attention or decorrelation strategies.

The remainder of this paper is organized as follows: Section 2 reviews related work in medical image classification, with a focus on deep learning methods, radiomics, and their integration, as well as the current state of research on attention mechanisms and feature decorrelation learning in this field. Section 3 details the proposed RadioGuide-DCN model architecture and its core modules, including the radiomics-guided mechanism, feature decorrelation loss, anti-attention fusion module, and the principles of the KAN classifier. Section 4 describes the experimental design, including datasets, preprocessing methods, evaluation metrics, and comparative approaches. Section 5 presents and analyzes the experimental results, comprehensively validating the performance advantages and effectiveness of each module of RadioGuide-DCN by comparison with multiple baseline methods. Section 6 summarizes the main work and innovations of this paper and discusses future research directions.

## 2. Related Work

### 2.1. Advances in Deep Learning for Medical Image Classification

Deep learning, especially convolutional neural networks (CNNs) like ResNet [7], DenseNet [8], and Inception [9], has significantly advanced medical image analysis, excelling in tumor detection, organ segmentation, and disease classification. For example, Xu et al. (2024) leveraged a CNN with multi-feature fusion (combining morphological and textural cues) to improve the classification of BI-RADS 4 breast ultrasound images, demonstrating higher accuracy than single-feature inputs [10]. However, CNNs are limited by their local receptive fields and struggle with capturing global context.

To overcome this, Transformer models with self-attention, such as ViT [11] and Swin Transformer [12], have been adopted for medical images, effectively modeling global information. For example, a transformer-based classification model reported accuracy of 86.7% and AUC of 0.95 on a breast ultrasound dataset [13], while a Swin U-Net + Cross Swin-T framework achieved 94.6% accuracy (AUC 96.2%) for referable diabetic retinopathy classification on EyePACS [14]. Self-supervised approaches (e.g., MAE, MiM) further enhanced ViT’s performance in unlabeled pre-training across twelve datasets [15]. Kolmogorov–Arnold Networks (KANs), such as MedKAN, which combine KANs with convolutional structures and specialized modules, have outperformed CNNs and Transformers on nine public medical datasets [16].

Despite these advances, medical imaging datasets are often small and annotated data scarce, while direct transfer from natural image pre-training (e.g., ImageNet) suffers from “domain shift” [17,18]. Domain-specific datasets like CPMID improve this, boosting accuracy by 4.30–8.86% over ImageNet pre-trained models [19].

### 2.2. Applications of Radiomics in Medical Image Analysis

Radiomics extracts high-throughput quantitative features—such as shape, intensity, and texture—from regions of interest (ROIs), and integrates these with machine learning algorithms for disease classification, prediction, and prognosis [20,21]. It has demonstrated substantial clinical utility in multiple malignancies, including lung and breast cancers, by capturing imaging biomarkers associated with tumor heterogeneity and treatment response [21]. Similarly, ultrasound-based radiomics has shown promise in predicting disease-free survival in triple-negative breast cancer [22].

However, traditional radiomics pipelines rely heavily on accurate ROI segmentation and manual feature engineering, resulting in limited robustness and high sensitivity to imaging variations [20]. In contrast, deep learning models enable automated feature extraction and hierarchical representation learning. For example, EfficientNetB6 achieved AUCs of 81.52% and 76.24% for breast microcalcification and mass classification, respectively, outperforming LDA-based radiomics approaches (66.9% and 61.5%) [23].

### 2.3. Fusion Strategies of Radiomics and Deep Learning

Fusing radiomics’ interpretability with deep learning’s feature learning is a major trend. Common approaches include input-, feature-, and decision-level fusion, with feature-level fusion (e.g., feature concatenation) most widely used. Tian et al. (2024) fused radiomics and ResNet-18 features, raising AUC for OLNM prediction in lung adenocarcinoma from 0.715/0.676 (single-modality) to 0.754 (fusion) [24]. Similarly, Sun et al. (2025) developed both early (feature-level) and late (decision-level) fusion models combining radiomics with 2D and 3D deep learning features, where the late fusion strategy achieved the best performance (AUC 89.8%) for differentiating invasive from microinvasive lung adenocarcinoma [25].

Yet, fusion models face challenges like feature heterogeneity, redundancy, and interpretability, particularly in multimodal scenarios where structural information can overshadow functional signals [6]. Efficient, end-to-end optimized fusion frameworks are needed.

### 2.4. The Importance of Feature Decorrelation and Representation Learning

High-dimensional imaging features often exhibit redundancy and collinearity, which can compromise model stability and generalization. Feature decorrelation techniques—such as orthogonal regularization or mutual information minimization—enhance independence among learned representations, particularly in limited or imbalanced medical datasets. By enforcing orthogonality, models can extract complementary rather than overlapping information from different feature sources, ultimately improving robustness and interpretability [26].

Recent studies have further incorporated these principles into multimodal medical image analysis. Some works introduce explicit decorrelation losses to reduce the correlation between handcrafted radiomic descriptors and deep features, facilitating complementary feature learning [27]. Others adopt mutual-information-based independence constraints to ensure that deep latent features remain informative yet non-redundant with respect to handcrafted radiomics [28]. Orthogonalization strategies have also been applied to encourage decorrelated subspaces between modalities, thereby reducing redundancy and improving discriminative capability [29]. More recent approaches leverage attention-based fusion to enhance the complementarity and stability of radiomic-deep representations [30].

Overall, feature decorrelation and representation learning jointly promote the development of stable, interpretable, and generalizable models. These strategies mitigate redundancy across heterogeneous feature spaces and enable networks to focus on disease-relevant patterns.

### 2.5. Summary

Beyond radiomics-guided frameworks, a wide range of deep learning architectures, including CNN-based models (e.g., VGG-style networks) and NAS-based approaches, have been explored for medical image classification in specific domains such as skin cancer and lung disease [31,32,33]. In parallel, system-level studies have investigated trustworthy and secure intelligent healthcare infrastructures, such as blockchain-enabled IoMT frameworks, to facilitate real-world deployment of medical imaging and decision-support systems [34]. While these studies reflect important progress in network architecture design and system integration, they typically do not explicitly focus on heterogeneous feature interaction and feature independence between handcrafted radiomics and deep representations. In contrast, our work emphasizes radiomics-guided feature interaction and decorrelation, which is complementary to both architecture-centric and system-centric advances.

In summary, while deep learning, radiomics, and their fusion have advanced medical image classification, challenges remain in integrating radiomics priors with deep features and improving feature independence. This study proposes RadioGuide-DCN, which utilizes radiomics-guided feature selection and decorrelation to achieve more informative, less redundant, and more discriminative representations, thereby enhancing classification performance.

## 3. Method

This paper proposes a novel deep learning framework named RadioGuide-DCN to enhance the accuracy and robustness of medical image classification. To address the heterogeneity of medical images, the subtlety of lesion features, and the demand for interpretability, RadioGuide-DCN deeply integrates radiomics features with deep learning features, harnessing the advantages of both to enable intelligent analysis of medical images.

### 3.1. Overall Framework: RadioGuide-DCN

**Figure 1:**
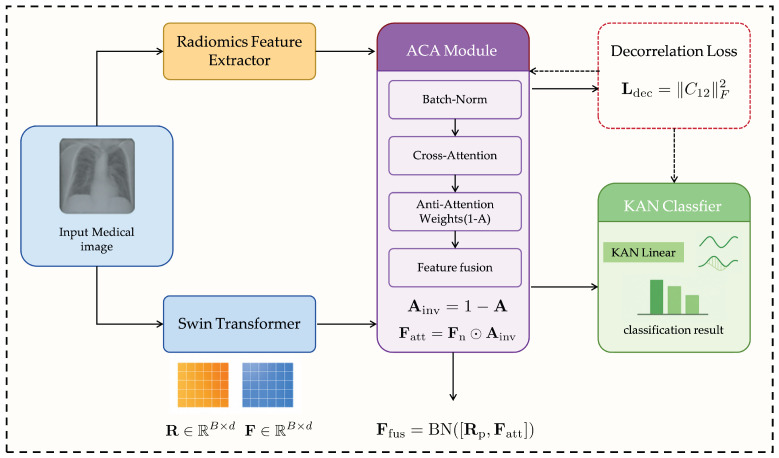
Overall architecture of the proposed RadioGuide-DCN framework.

The proposed RadioGuide-DCN is a multi-stage deep learning framework that integrates interpretable radiomics features with expressive deep learning features to improve the accuracy and robustness of medical image classification. Its modular architecture performs efficient feature fusion and decorrelation, enhancing discriminative capability, reducing redundancy, and improving generalizability, as illustrated in Figure 1.

#### 3.1.1. Core Modules

Anti-Cross-Attention (ACA) Module. ACA introduces a reverse attention mechanism that amplifies complementary and discriminative information between cross-modal features. Unlike traditional attention emphasizing high-correlation dimensions, ACA focuses on low-correlation yet informative components, uncovering valuable information often overlooked by standard methods.

Decorrelation Loss Module. Serving as a regularizer, this module explicitly minimizes linear correlations among fused features to promote feature independence. It reduces redundancy, increases feature-space efficiency, and enhances model adaptability to unseen data.

Kolmogorov–Arnold Network (KAN) Classifier. Inspired by the Kolmogorov–Arnold representation theorem, the KAN classifier replaces fixed node activations with learnable edge-based functions (e.g., B-splines), enabling flexible nonlinear modeling and improving interpretability and generalization [35].

#### 3.1.2. Processing Workflow

1. Feature Extraction: Radiomics and deep learning models (e.g., ResNet, Swin Transformer) extract complementary handcrafted and semantic features.
2. ACA Fusion: The ACA module fuses both feature sets, emphasizing low-correlation yet discriminative dimensions.
3. Decorrelation Optimization: A decorrelation loss enforces feature independence, improving representational expressiveness.
4. KAN Classification: The optimized features are classified via KAN, leveraging its nonlinear modeling strength.

#### 3.1.3. Framework Logic and Contribution

RadioGuide-DCN progressively refines feature representations: ACA determines what to fuse, the decorrelation mechanism specifies how to fuse, and KAN defines how to classify. This architecture systematically addresses feature heterogeneity, redundancy, and limited adaptability in multimodal medical imaging, achieving superior accuracy and generalizability across diverse datasets.

### 3.2. Feature Extraction

High-quality feature extraction is fundamental to medical image classification. To fully leverage the complementarity of multi-source features, this study acquires and preprocesses features from both deep learning and radiomics perspectives, aiming to capture the multi-level information contained in medical images.

#### 3.2.1. Deep Learning Feature Extraction

After standard preprocessing (e.g., normalization and data augmentation), medical images are input into deep learning models to extract high-level semantic representations. The framework employs flexible backbones such as the Swin Transformer and Convolutional Neural Networks (CNNs). The Swin Transformer, with its hierarchical architecture and shifted-window self-attention, effectively captures multi-scale spatial dependencies and complex contextual patterns, while CNNs learn complementary hierarchical features from local textures to global semantics.

In this study, the Swin Transformer encoder outputs a deep feature vector denoted as $F_{n}$ with an original dimensionality of 768. To align with radiomics features and reduce model complexity, $F_{n}$ is linearly mapped to a unified 512-dimensional space, preserving discriminative power while improving fusion efficiency and generalizability [12].

#### 3.2.2. Radiomics Feature Extraction and Selection

Radiomics feature extraction is based on region of interest (ROI) segmentation, which may be manual, semi-automatic, or fully automatic. For images lacking ROI masks, features are extracted from the entire image. Following IBSI standards, a comprehensive set of radiomic features is initially obtained, including: (1) Shape features (e.g., volume, surface area, sphericity); (2) First-order statistics (e.g., mean, standard deviation, skewness, kurtosis); and (3) Texture features describing tissue heterogeneity, derived from GLCM, GLRLM, GLSZM, NGTDM, and GLDM matrices.

The raw radiomic feature set $R^{orig}$ typically exceeds 100 dimensions. To retain the most informative and discriminative features while preventing overfitting, LASSO regression is applied for embedded feature selection. After selection, 100 key features are preserved and denoted as *R*. To ensure dimensional consistency with the deep feature $F_{n}$ and facilitate subsequent fusion, *R* is linearly projected into a 512-dimensional space.

### 3.3. Anti-Cross-Attention Module (ACA)

In medical image analysis, effectively fusing heterogeneous features (e.g., radiomics and deep learning) to exploit their complementarity while suppressing redundancy is crucial for enhancing a model’s discriminative power and generalization. Traditional attention mechanisms, including standard cross-attention [36], typically emphasize feature dimensions that exhibit high cross-modal correlation or similarity during multimodal fusion. However, in complex medical imaging data, discriminative cues crucial for classification may often reside in low-correlation dimensions that offer unique or complementary information not captured by highly correlated features. Solely emphasizing such highly correlated dimensions may thus lead to the suppression of these informative but less correlated signals.

**Figure 2:**
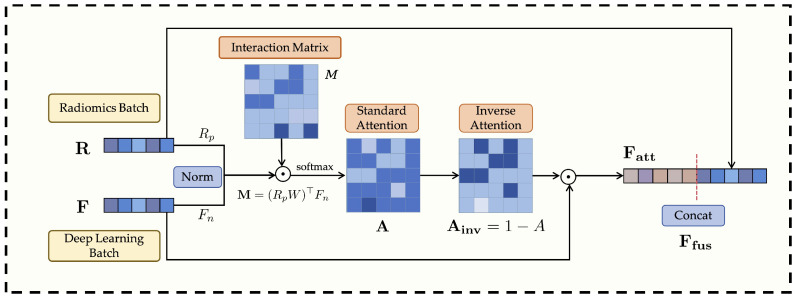
Overall architecture of the Anti-Cross-Attention (ACA) module.

To address this limitation, this paper proposes a novel Anti-Cross-Attention (ACA) mechanism. The core idea of ACA is to invert the conventional attention paradigm—actively emphasizing those feature dimensions that receive low attention scores (i.e., less correlated regions) but potentially carry highly complementary and discriminative information. In this way, ACA enables a more comprehensive exploitation of latent multimodal complementarity, thereby enhancing both the discriminative power and generalizability of the fused representations. The structure of this module is illustrated in Figure 2.

Suppose after feature extraction and alignment, the batch of radiomics features is represented as $R\in R^{B\times d}$ and the deep learning features as $F\in R^{B\times d}$, where *B* is the batch size and *d* is the unified feature dimension (e.g., 512 in this study). To ensure numerical stability in subsequent interactions and eliminate scale biases between modalities, both feature groups are first normalized. The radiomics features *R* may be projected through a learnable linear transformation $\left(W_{\mathrm{rp}},b_{\mathrm{rp}}\right)$ if their original dimension differs from *d* or requires further transformation, and then subjected to $L_{2}$ normalization. The deep learning features *F* are also $L_{2}$ normalized. The normalized features are denoted as $R_{p}$ and $F_{n}$:

(1) $$R_{p}=\mathrm{Norm}\left(W_{\mathrm{rp}}R+b_{\mathrm{rp}}\right),\;F_{n}=\mathrm{Norm}\left(F\right)$$

where Norm(·) denotes the $L_{2}$ normalization operation.

Next, the cross-modal interaction between the normalized features is modeled through a bilinear mapping, as formulated in Equations (2) and (3). For each sample *b* in the batch, the interaction matrix $M_{b}\in R^{d\times d}$ is computed by measuring the pairwise relationships between the projected radiomics features $r_{p,b}$ and the deep features $f_{n,b}$:

(2) $$M_{b,ij}=\left(\sum_{k=1}^{d}r_{p,b,k}W_{k,i}\right)f_{n,b,j}$$

where $W\in R^{d\times d}$ is a learnable bilinear transformation matrix that captures the channel-level correspondence between the two modalities. In compact matrix form, Equation (2) can be expressed as

(3) $$M_{b}={\left(r_{p,b}W\right)}^{⊤}f_{n,b}$$

which yields a full pairwise correlation map across feature dimensions.

The resulting interaction tensor *M* is then batch-normalized and converted into attention weights through a softmax operation, as shown in Equation (4):

(4) $$M_{\mathrm{bn}}=\mathrm{BN}\left(M\right),\;A={\mathrm{softmax}}_{\dim=2}\left(M_{\mathrm{bn}}\right)$$

where BN(·) denotes the batch normalization operation, and the softmax is applied along the feature dimension to obtain normalized attention weights for each modality pair.

To highlight weakly correlated yet potentially discriminative cross-modal feature pairs, the proposed Anti-Cross-Attention (ACA) mechanism introduces an inverse attention weighting strategy, formulated as

(5) $$A_{\mathrm{inv}}=1-A$$

where higher weights are assigned to feature dimensions that receive lower attention scores in the standard cross-attention map. To prepare this d×d matrix for element-wise application, we first aggregate it along the radiomics feature dimension (column sum) to produce a 1×d attention vector. A learnable residual gating mitigates potential gradient vanishing introduced by pure inverse attention and adaptively re-balances feature weighting, ensuring stable optimization and flexible feature interaction.

The inverse attention weights are then applied to reweight the normalized deep features, emphasizing complementary information, followed by feature concatenation and batch normalization for fusion:

(6) $$F_{\mathrm{att}}=F_{n}⊙A_{\mathrm{inv}}$$

(7) $$F_{\mathrm{fus}}=\mathrm{BN}\left(\mathrm{Concat}(R_{p},\;F_{\mathrm{att}})\right)$$

where ⊙ denotes element-wise multiplication, and Concat(·) represents the concatenation operation along the feature dimension. As formulated in Equations (5)–(7), the Anti-Cross-Attention mechanism explicitly emphasizes low-correlation yet complementary information across modalities, thereby enhancing the diversity and robustness of the fused feature representations.

### 3.4. Decorrelation Loss Module

**Figure 3:**
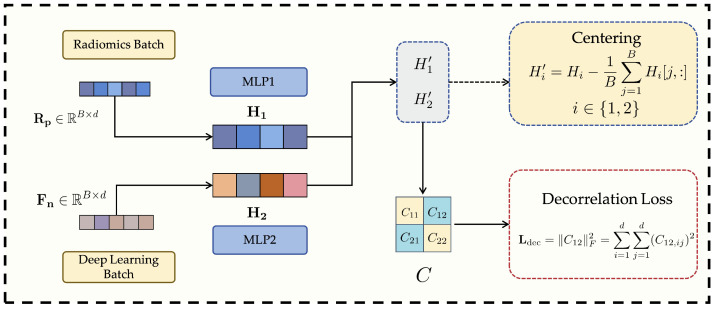
Structure of the proposed Decorrelation Loss Module.

Although the Anti-Cross-Attention (ACA) mechanism effectively enhances the complementarity between radiomics features and deep learning features, the fused multi-source features (or their respective representations prior to fusion) may still exhibit a certain degree of linear redundancy. Such redundancy not only reduces the effective utilization of the feature space but may also exacerbate model overfitting, thereby impairing generalization to unseen data. To address this issue, this study introduces a decorrelation loss as a regularization constraint during model training. The purpose of this loss function is to encourage the model to learn more independent and complementary feature representations between components (e.g., features from radiomics and those from deep learning), thereby improving the overall discriminative power and robustness of the model. This strategy is consistent with prior studies that adopt decorrelation approaches to eliminate redundancy, enhance feature quality, or improve model performance. The structure of this module is illustrated in Figure 3.

In terms of implementation, we assume two sets of feature views, denoted as $H_{1},\;H_{2}\in R^{B\times d}$, where *B* is the batch size and *d* is the feature dimension. These two feature views can be interpreted as follows: when computing the decorrelation loss [37], the normalized radiomics features $R_{p}$ and deep learning features $F_{n}$ defined in Equation (1) are mapped to the same *d*-dimensional latent space via their respective multilayer perceptrons (MLPs), i.e., ${\mathrm{MLP}}_{1}$ and ${\mathrm{MLP}}_{2}$, yielding $H_{1}={\mathrm{MLP}}_{1}\left(R_{p}\right)$ and $H_{2}={\mathrm{MLP}}_{2}\left(F_{n}\right)$. The objective of the decorrelation loss is to ensure that these two parallel latent representations, $H_{1}$ and $H_{2}$, are as linearly independent as possible.

First, both sets of feature views are centered by subtracting the mean along each feature dimension, thus eliminating mean shift effects on subsequent correlation computations:

(8) $$H_{i}^{\prime }=H_{i}-\frac{1}{B}\sum_{j=1}^{B}H_{i}\left[j,:\right],\;i\in \left\{1,2\right\}$$

where $H_{i}\left[j,:\right]$ denotes the feature vector of the *j*-th sample in the *i*-th feature view.

Next, a joint covariance matrix $C\in R^{2d\times 2d}$ is constructed to comprehensively measure both the intra- and inter-view linear correlations between the two centered feature views. This covariance matrix consists of four d×d sub-blocks:

- The diagonal blocks $C_{11}=\frac{1}{B-1}H_{1}^{\prime ⊤}H_{1}^{\prime }+\epsilon I$ and $C_{22}=\frac{1}{B-1}H_{2}^{\prime ⊤}H_{2}^{\prime }+\epsilon I$ represent the within-view covariance matrices of $H_{1}^{\prime }$ and $H_{2}^{\prime }$, respectively, where ϵI is a small regularization term (ϵ is a small positive constant, and *I* is the identity matrix) to ensure numerical stability and positive semi-definiteness.
- The off-diagonal blocks $C_{12}=\frac{1}{B-1}H_{1}^{\prime ⊤}H_{2}^{\prime }$ and $C_{21}=H_{2}^{\prime ⊤}H_{1}^{\prime }=C_{12}^{⊤}$ represent the cross-covariance matrices between $H_{1}^{\prime }$ and $H_{2}^{\prime }$.

The complete covariance matrix *C* can be expressed as:

(9) $$C=\left(\begin{array}{cc} C_{11} & C_{12}\\ C_{21} & C_{22} \end{array}\right)$$

To effectively measure and penalize the linear correlation between the two latent feature views, we employ a decorrelation loss based on the cross-covariance matrix $C_{12}$. Instead of relying on the eigenvalues of the full covariance matrix, which primarily reflect total variance rather than inter-view redundancy, we directly minimize the Frobenius norm of the cross-covariance block as follows:

(10) $$L_{\mathrm{dec}}={∥C_{12}∥}_{F}^{2}=\sum_{i=1}^{d}\sum_{j=1}^{d}{\left(C_{12,ij}\right)}^{2}$$

where $C_{12}=\frac{1}{B-1}H_{1}^{\prime ⊤}H_{2}^{\prime }$ measures the linear dependence between the centered feature views $H_{1}^{\prime }$ and $H_{2}^{\prime }$. By minimizing $L_{\mathrm{dec}}$, the network explicitly suppresses cross-modal linear correlations, thereby encouraging statistical independence and reducing redundancy between radiomics and deep representations [38]. This explicit decorrelation regularization is the core function of the proposed module.

During model training, to balance the classification objective and the optimization of feature disentanglement, the final training objective is defined as the weighted sum of the standard cross-entropy loss ($L_{\mathrm{ce}}$) and the decorrelation loss ($L_{\mathrm{dec}}$):

(11) $$L_{\mathrm{tot}}=L_{\mathrm{ce}}+\alpha \cdot L_{\mathrm{dec}}$$

Here, $L_{\mathrm{ce}}$ ensures the model’s basic classification capability; $L_{\mathrm{dec}}$ promotes the independence and diversity of the fused features (or their source representations), reducing linear redundancy; and α is the weighting coefficient for the decorrelation loss, controlling its contribution to the total loss.

It is noteworthy that the weighting coefficient α is not kept constant during training but follows an incremental scheduling strategy. Specifically, its value increases gradually with the number of training epochs, for instance:

$$\alpha =\alpha_{\max}\times \frac{\mathrm{current}\_\mathrm{epoch}}{N_{\mathrm{total}}\_\mathrm{epochs}},$$

where $\alpha_{\max}$ is the preset maximum weight and $N_{\mathrm{total}}\_\mathrm{epochs}$ is the total number of epochs.

This dynamic adjustment offers three practical advantages:

1. Stable early training: A small initial α allows the optimization to focus on the primary classification objective, ensuring rapid convergence and stable feature learning.
2. Progressive feature independence: As training proceeds, the gradual increase of α strengthens decorrelation regularization, encouraging complementary and non-redundant representations from different feature sources.
3. Balanced optimization: The incremental schedule maintains a dynamic balance between classification accuracy and feature disentanglement, avoiding excessive regularization.

It should be clarified that the incremental scheduling of the decorrelation loss weight is adopted as a practical optimization strategy to stabilize training, rather than being claimed as a methodological contribution. Different from conventional covariance-based regularizers that indiscriminately suppress correlations within a single feature space, the proposed decorrelation term is applied across heterogeneous radiomics-deep latent representations after guided feature alignment. This design aims to reduce redundant cross-modal dependencies while preserving complementary low-correlation discriminative information, thereby facilitating more effective heterogeneous feature fusion.

Through this strategy, the model prioritizes classification accuracy in the early phase and enhances feature independence in later stages. The ACA module focuses on mining complementary information (what to fuse), while the decorrelation loss refines representation independence (how to represent). Together, they jointly improve the quality and generalization of fused features. Extensive experiments confirm that this dual optimization mechanism significantly enhances the robustness and discriminative power of the final classification model in complex medical imaging scenarios.

### 3.5. Kolmogorov–Arnold Network Classifier (KAN Classifier)

In the RadioGuide-DCN framework, the final classification task is performed on the refined fused feature vector $F_{\mathrm{fus}}$, which has already incorporated complementary radiomics-deep features through ACA and been further purified by decorrelation loss. Such a heterogeneous and structurally complex representation requires a classifier capable of modeling high-order nonlinear relationships with stability and efficiency. To this end, we employ the Kolmogorov–Arnold Network (KAN) [35] as the final classification head. Unlike conventional MLPs that rely on fixed node-wise activations (e.g., ReLU), KAN places learnable spline-based activation functions on network edges, enabling flexible functional mappings that adaptively capture nonlinear behaviors across feature dimensions. This design is particularly advantageous for multimodal medical features, which often exhibit non-Gaussian distributions and localized nonlinear patterns. Moreover, the effectiveness of KAN-like architectures in medical imaging has been supported by recent evidence: MedKAN [16] combines KAN structures with convolutional encoders and achieves superior performance over CNN and Transformer baselines across multiple public medical datasets, demonstrating its robustness under small-sample and heterogeneous data conditions. Within our framework, the refined feature space produced by ACA and decorrelation loss provides an ideal input domain for KAN, allowing it to form accurate and stable decision boundaries. In addition, the spline functions learned by KAN are explicitly visualizable, offering transparent insights into how different fused feature dimensions contribute to the final prediction—an important property for clinically trustworthy AI systems. Although its computational cost is slightly higher than that of an MLP, the substantial gains in nonlinear modeling capacity, robustness, and interpretability make KAN a well-justified choice for the final classifier in RadioGuide-DCN.

Consistent with recent findings in deep radiomics studies, our ablation results indicate that the primary performance gains of the proposed framework arise from improved feature fusion and decorrelation rather than the choice of classifier. The KAN classifier is therefore not an essential component of the proposed method, but serves as a compatible nonlinear classifier that can better exploit the decorrelated heterogeneous feature representations. Other standard classifiers can be readily integrated into the proposed framework with comparable performance trends.

## 4. Experimental Design

To comprehensively evaluate the performance of the proposed RadioGuide-DCN framework in medical image classification, this section introduces the datasets, evaluation metrics, implementation details, and ablation studies.

### 4.1. Datasets

**Figure 4:**
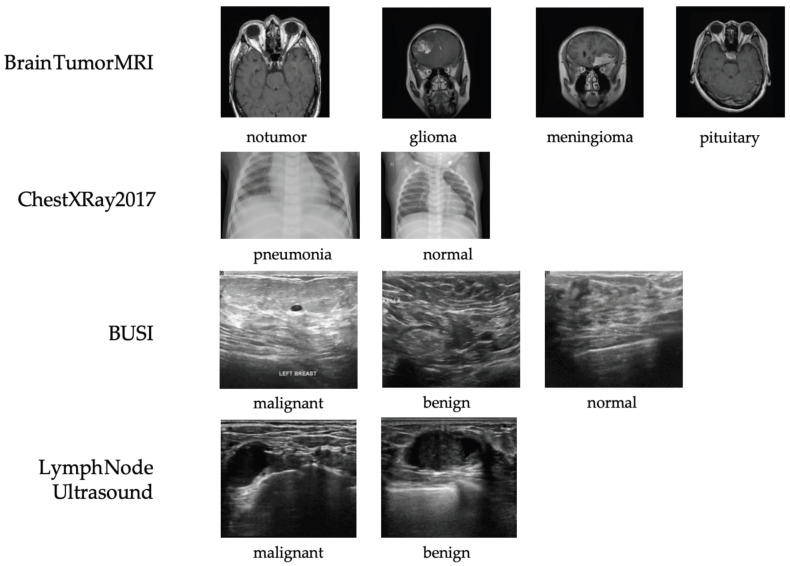
Representative samples from each dataset used in this study, including X-ray (ChestXRay2017), ultrasound (BUSI and private lymph node ultrasound), and MRI (Brain Tumor MRI). All images shown are randomly selected samples from the corresponding datasets and are used solely for illustrative purposes. No images are reproduced from external publications.

Four representative datasets from different imaging modalities—X-ray, ultrasound, and MRI—were used to evaluate the proposed framework (see Figure 4). Three are publicly available, and one is a private clinical dataset constructed by our team.

- ChestXRay2017: NIH chest X-ray dataset containing 5856 images (subset used) labeled as normal, bacterial pneumonia, or viral pneumonia. It provides a large-scale benchmark for thoracic disease classification but includes moderate label noise due to NLP-based annotation [39].
- BUSI: Breast ultrasound dataset with 780 images from 600 patients, categorized as normal, benign, or malignant. It captures the complexity of ultrasound imaging for breast lesion analysis [40].
- Brain Tumor MRI: Dataset of 7023 MRI images across four categories (glioma, meningioma, pituitary tumor, and normal). It supports multi-class tumor detection research [41].
- Private Lymph Node Ultrasound: Self-collected clinical dataset with 1038 annotated cases (473 benign, 565 malignant), reviewed by radiologists and approved by the IRB to ensure quality and compliance.

Representative samples and class distributions are shown in Figure 4, and dataset statistics are summarized in Table 1.

To enhance generalization and mitigate overfitting, online data augmentation was applied during training, including random horizontal flipping, rotation ($\pm 15^{\circ }$), scaling (0.8–1.2), and brightness/contrast adjustment. Augmentation parameters were optimized per modality to maintain clinical realism and avoid artifacts.

Taken together, the datasets used in this study cover three representative medical imaging modalities with distinct physical principles and visual characteristics, including X-ray (ChestXRay2017), ultrasound (BUSI and private lymph node ultrasound), and MRI (Brain Tumor MRI). These modalities differ substantially in image appearance, noise patterns, contrast mechanisms, and anatomical focus. Evaluating the proposed RadioGuide-DCN framework across such heterogeneous modalities enables a comprehensive assessment of its generalizability and supports our claim that the proposed method is applicable to a broad range of medical image classification tasks rather than being limited to a single imaging modality.

### 4.2. Evaluation Metrics

To comprehensively evaluate the performance of RadioGuide-DCN and comparative methods in medical image classification, this study adopts four mainstream classification metrics, which collectively account for accuracy, robustness, and clinical significance.

Accuracy (Acc): Measures the overall ability of the model to correctly classify all samples. The calculation is as follows:

(12) $$\mathrm{Acc}=\frac{TP+TN}{TP+TN+FP+FN}$$

where TP and TN denote the numbers of correctly identified positive and negative cases, respectively, and FP and FN are the numbers of incorrectly classified cases.

Area Under the ROC Curve (AUC): AUC measures the model’s ability to distinguish between positive and negative samples. A value closer to 1 indicates better performance. Notably, when dealing with class imbalance, AUC is more robust than accuracy.

Sensitivity (Sen): Also known as recall, sensitivity represents the proportion of actual positive cases correctly identified:

(13) $$\mathrm{Sen}=\frac{TP}{TP+FN}$$

Specificity (Spe): Also known as the True Negative Rate (TNR), specificity measures the proportion of actual negative samples that are correctly identified as negative. It is defined as:

(14) $$\mathrm{Spe}=\frac{TN}{TN+FP}$$

### 4.3. Implementation Details

All datasets were randomly split into training and testing sets with an 8:2 ratio, maintaining balanced class distributions. Models were trained for 200 epochs with a batch size of 32. We employed early stopping to prevent overfitting, terminating training if the validation performance did not improve within a fixed patience window. Unless otherwise specified, all models were optimized using the Adam optimizer with an initial learning rate of $1\times 10^{-4}$ and a weight decay of $1\times 10^{-5}$. The batch size was fixed to 32 for all experiments. The maximum weight of the decorrelation loss was set to $\alpha_{\max}=0.2$, and its value was gradually increased during training following the scheduling strategy described in Section 3.4. The fused feature dimension was fixed to 512. For the KAN classifier, the spline grid size was set to 5 and the spline order was set to 3. All experiments were conducted under the same training protocol to ensure fair comparison across different methods.

Comparative Baselines. To comprehensively evaluate the proposed RadioGuide-DCN, we compared it against representative medical image classification methods from different architectural paradigms:

- CNN-based: ResNet-50, a classical convolutional baseline for visual recognition tasks.
- Transformer-based: ViT-S/16, ViT-B/16 [11], and Swin Transformer-S/B [12], which model global dependencies via self-attention and have shown strong performance in medical imaging.
- Mamba-based: MedMamba-T and MedMamba-B [42], recent State Space Model (SSM) architectures that efficiently capture long-range spatial relationships.
- Radiomics-based: Classical machine learning models (SVM, XGBoost [43], and Random Forest) trained on standardized radiomic features extracted using PyRadiomics (version 2.0.1).

To ensure fair comparison, all methods used the same dataset partitions, data augmentation strategies, and evaluation metrics as RadioGuide-DCN. This unified experimental protocol isolates architectural contributions and ensures reproducibility across models.

### 4.4. Ablation Study

To evaluate the contribution of each core component in RadioGuide-DCN, we conducted systematic ablation experiments on the BUSI breast ultrasound dataset. Each module was selectively removed or replaced to quantify its impact on classification performance.

(1) Feature Fusion Baseline (Swin + Radiomics + FC). Deep semantic features extracted by the Swin Transformer were concatenated with radiomic features, followed by a fully connected (FC) classifier. This serves as the standard multimodal fusion baseline.

(2) + ACA and Decorrelation Loss (Swin + Radiomics + ACA + Deco + FC). Building upon the baseline, the Anti-Cross Attention (ACA) module and feature decorrelation loss were introduced. ACA enhances cross-modal interaction by emphasizing complementary information, while the decorrelation loss reduces redundancy and improves feature diversity.

(3) + KAN Classifier (Swin + Radiomics + ACA + Deco + KAN). Finally, the FC layer was replaced with a Kolmogorov–Arnold Network (KAN) classifier to improve nonlinear modeling and interpretability. KAN further strengthens the discriminative capacity of the fused representations.

All ablation settings followed the same training protocol, evaluation metrics (Accuracy, AUC, Sensitivity, Specificity), and 80/20 data split as in the main experiments. The results, discussed in Section 5, demonstrate that each component—ACA, decorrelation loss, and KAN—provides consistent and complementary performance gains, validating the effectiveness of the proposed design.

## 5. Results

### 5.1. Overall Performance Comparison

#### 5.1.1. BUSI Dataset

The reported accuracy of 93.63% should be interpreted in comparison with representative baseline methods evaluated on the same datasets, where the proposed approach consistently demonstrates superior or competitive performance rather than relying on an absolute accuracy threshold. Table 2 summarizes the performance of all comparative methods on the BUrsT Ultrasound (BUSI) dataset. Our proposed RadioGuide-DCN achieves the best results across all evaluation metrics, reaching an accuracy of 93.63% and an AUC of 0.9853. Compared with the strongest transformer baseline (Swin-B), RadioGuide-DCN yields gains of +3.8% in accuracy and +0.0055 in AUC, demonstrating the superior discriminative capability of our dual-modality design. Furthermore, the model attains both high sensitivity (94.55%) and specificity (96.33%), indicating robust lesion detection performance and reduced false positives—key aspects for reliable clinical decision support. These results confirm that integrating radiomic priors with deep contextual representations effectively enhances feature complementarity and generalization in ultrasound image classification. Table 2 summarizes the performance of all methods compared in the BUrsT ultrasound (BUSI) dataset. The best values in each column are highlighted in bold.

**Figure 5:**
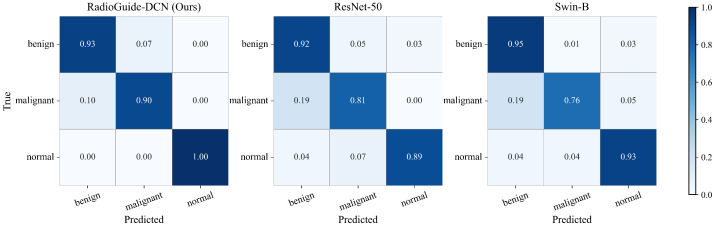
Comparison of normalized confusion matrices on the BUSI test set among RadioGuide-DCN (ours), ResNet-50, and Swin-B. RadioGuide-DCN achieves higher class separability and lower inter-class confusion, particularly between benign and malignant lesions.

To further illustrate the classification behavior of different models on the BUSI test set, Figure 5 presents the normalized confusion matrices of RadioGuide-DCN, ResNet-50, and Swin-B. It can be observed that RadioGuide-DCN exhibits the strongest diagonal dominance, indicating more accurate predictions across all categories and fewer misclassifications between benign and malignant lesions. In contrast, the baselines show higher confusion particularly between these two classes, demonstrating that the proposed dual-modality design enables more robust and reliable discrimination.

#### 5.1.2. Other Datasets

Table 3 summarizes the quantitative results on the Private Lymph Node Ultrasound dataset. Overall, our proposed RadioGuide-DCN achieves the highest performance across all evaluation metrics, with an accuracy of 92.75% and an AUC of 0.9753, surpassing the strongest baseline (Swin-B) by 2.3% and 0.0078 in accuracy and AUC, respectively. Notably, RadioGuide-DCN also attains balanced sensitivity (92.04%) and specificity (93.62%), indicating both low false negative and false positive rates—an essential property for clinical ultrasound applications. Compared to traditional radiomics-based classifiers (SVM, XGBoost, Random Forest), the hybrid deep-radiomics architecture demonstrates superior discriminative capacity and robustness, validating the benefit of the proposed multimodal feature fusion and nonlinear KAN-based classification. These results indicate that the proposed framework maintains stable performance improvements not only in terms of overall accuracy but also in clinically relevant metrics such as sensitivity and specificity. The consistent advantage over both deep learning baselines and traditional radiomics classifiers suggests that the proposed radiomics-guided fusion strategy effectively captures complementary information in ultrasound imaging, which is known to be noisy and operator-dependent. This robustness further supports the applicability of the proposed method in real-world clinical scenarios.

As shown in Table 4, RadioGuide-DCN consistently achieves the best performance across both X-ray and MRI modalities. On ChestXRay2017, our model surpasses the best transformer-based baseline (Swin-B) by 1.6% in accuracy and 0.013 in AUC. Similarly, on Brain Tumor MRI, RadioGuide-DCN improves AUC to 0.993 and achieves 98.3% accuracy, indicating excellent generalization across distinct imaging modalities. Table 4 further demonstrates the cross-modality generalization capability of the proposed framework across fundamentally different imaging modalities. On the ChestXRay2017 dataset, which is based on projection imaging, RadioGuide-DCN consistently outperforms CNN- and Transformer-based baselines, indicating effective feature learning under limited depth and contrast conditions. On the Brain Tumor MRI dataset, which exhibits high soft-tissue contrast and complex anatomical structures, the proposed method achieves near-saturated performance with the highest accuracy and AUC among all compared methods. These results suggest that the proposed radiomics-guided fusion and decorrelation strategy generalizes well across imaging modalities with substantially different physical principles, contrast mechanisms, and anatomical characteristics.

#### 5.1.3. Training Dynamics and Model Stability

**Figure 6:**
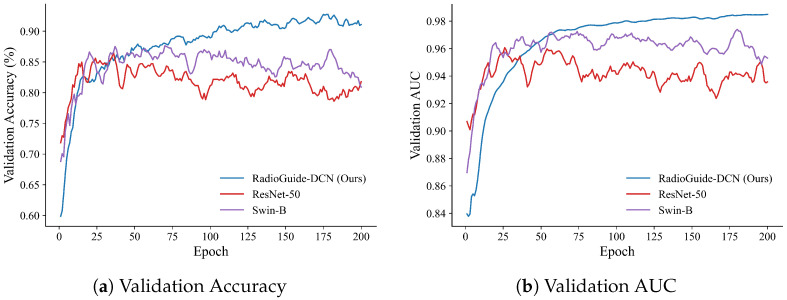
Validation performance on the BUSI dataset. (**a**) Validation accuracy curves of RadioGuide-DCN, Swin-B, and ResNet-50, where RadioGuide-DCN achieves consistently higher accuracy with reduced oscillations. (**b**) Validation AUC curves showing that RadioGuide-DCN maintains the highest and most stable AUC throughout training, evidencing robust discriminative learning.

**Figure 7:**
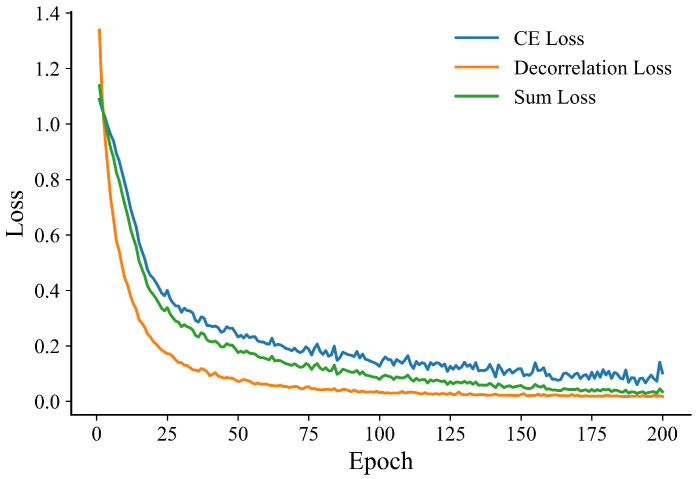
Training loss evolution of RadioGuide-DCN on the BUSI dataset.

To investigate the optimization behavior and generalization stability of different models, we further analyze the training dynamics on the BUSI dataset. As shown in Figure 6 and Figure 7, RadioGuide-DCN converges faster and achieves higher validation metrics with lower variance compared to Swin-B and ResNet-50, reflecting superior optimization stability and stronger generalization capability.

From the validation metrics, RadioGuide-DCN exhibits rapid early-epoch convergence and minimal late-epoch oscillations, suggesting that the Anti-Cross-Attention and decorrelation regularization provide stronger gradient guidance and smoother optimization trajectories.

### 5.2. Ablation Study Results

We conduct ablation studies on the BUSI dataset under identical training protocols. Table 5 presents the ablation results on the BUSI dataset. Starting from the Baseline (Swin features + radiomics + FC), adding ACA provides a small improvement over the Baseline (+0.78% Acc and +0.0015 AUC). Introducing the Deco loss yields the largest single-step gain, raising performance to 92.90% Acc and 0.9839 AUC (+2.02% Acc and +0.0074 AUC compared to ACA). Replacing the FC with a KAN classifier brings the final incremental boost (+0.73% Acc and +0.0014 AUC), resulting in the best overall performance. The ablation results presented in Baseline reveal the contribution of each core component of the proposed RadioGuide-DCN framework. Introducing the Anti-Cross-Attention (ACA) module provides a moderate performance improvement by enhancing complementary feature interaction across modalities. Incorporating the decorrelation loss yields the most significant performance gain, highlighting the importance of explicitly reducing cross-modal feature redundancy. Replacing the fully connected classifier with the KAN classifier results in an additional but relatively smaller improvement, suggesting that the primary performance gains stem from improved feature representation quality rather than classifier complexity. Furthermore, as illustrated in Figure 6 and Figure 7, RadioGuide-DCN exhibits faster convergence and reduced oscillations in both validation performance and training loss compared to baseline models. This stable optimization behavior indicates that the proposed fusion and decorrelation mechanisms provide more informative gradient guidance and improved training stability, contributing to better generalization.

## 6. Discussion

### 6.1. Overview of the Proposed Method

The proposed RadioGuide-DCN introduces a radiomics-guided, decorrelated deep classification framework that bridges the interpretability of traditional radiomics with the expressive power of deep neural networks. Unlike conventional medical image classifiers that rely solely on convolutional or Transformer-based representations, our approach explicitly incorporates radiomics priors as domain knowledge and designs an anti-cross-attention (ACA) mechanism to exploit the low-correlation yet highly discriminative components across modalities. This design allows the model to highlight complementary information between handcrafted and learned features that would otherwise be suppressed in conventional attention schemes. Additionally, the introduction of a decorrelation loss acts as a structural regularizer, reducing linear dependencies among features and improving generalization to unseen imaging data. Finally, the Kolmogorov–Arnold Network (KAN) classifier provides a nonlinear, spline-based transformation that enhances interpretability while maintaining high discriminative performance.

Although an X-ray image is used as an illustrative example in Figure 1, the proposed RadioGuide-DCN framework is not limited to a single imaging modality. Extensive experiments on heterogeneous datasets demonstrate that the proposed method generalizes well across multiple medical imaging modalities, including X-ray, ultrasound, and MRI. This cross-modality applicability highlights the potential of the proposed framework for a wide range of medical image classification tasks in real clinical scenarios.

Together, these components form a coherent and modular framework capable of capturing both global semantic patterns and fine-grained textural variations. The combination of interpretability (from radiomics), representation flexibility (from ACA and decorrelation), and nonlinear adaptability (from KAN) positions RadioGuide-DCN as a robust, generalizable paradigm for multimodal medical image classification.

### 6.2. Method Evaluation

Comprehensive experiments across four imaging modalities—X-ray, ultrasound, and MRI—demonstrate that RadioGuide-DCN consistently outperforms existing CNN-, Transformer-, and radiomics-based baselines. On the BUSI ultrasound dataset, the proposed framework achieves an accuracy of 93.63% and an AUC of 0.9853, surpassing the strongest baseline (Swin-B) by +3.8% in accuracy and +0.0055 in AUC. Similar improvements are observed on the private lymph node dataset (+2.3% accuracy, +0.0078 AUC) and the ChestXRay2017 and Brain Tumor MRI datasets, where RadioGuide-DCN achieves the highest overall results among all compared models.

Ablation studies confirm the effectiveness of each module. The decorrelation loss mechanism yields the largest single performance gain (+2.02% accuracy), indicating that the explicit modeling of cross-modal complementarity plays a critical role in feature fusion. The decorrelation loss further enhances feature independence, leading to better generalization, especially in small-sample or high-heterogeneity datasets. The adoption of KAN as the final classifier provides an additional improvement, suggesting that spline-based nonlinear mapping can better model complex decision boundaries compared to fixed-activation MLPs.

In addition to quantitative metrics, qualitative analyses show that ACA improves attention localization to diagnostically relevant regions (e.g., tumor boundaries, lesion texture), while the decorrelation constraint suppresses spurious correlations and improves robustness to imaging variations such as illumination or contrast. These findings collectively demonstrate that RadioGuide-DCN not only achieves superior accuracy but also enhances interpretability and stability—two essential properties for clinical deployment.

### 6.3. Limitations and Future Work

Despite the strong empirical performance and generalizability, several limitations remain. First, the current framework depends on accurate region-of-interest (ROI) segmentation for radiomics feature extraction, which may limit scalability in fully automated pipelines. Future work will explore integrating automatic segmentation and self-supervised representation learning to minimize human intervention. Second, although the decorrelation loss effectively reduces redundancy, its computation relies on batch-wise covariance estimation, which introduces non-negligible additional training overhead. Lightweight approximations or stochastic decorrelation strategies could further improve efficiency for large-scale datasets. Third, while the KAN classifier enhances nonlinear modeling and interpretability, its interpretive visualization remains relatively unexplored; future research will investigate mapping learned spline functions back to semantic image features for explainable AI in radiology.

Moreover, expanding the RadioGuide-DCN framework toward multimodal integration (e.g., combining imaging with clinical or genomic data) and few-shot learning scenarios will further enhance its clinical applicability. Another promising direction is the development of an end-to-end differentiable radiomics extractor that jointly optimizes radiomics and deep representations within a unified architecture. We believe that continued exploration along these directions will further establish RadioGuide-DCN as a versatile and interpretable foundation for next-generation intelligent medical image analysis systems.

## Figures and Tables

**Table 1:** Summary of datasets used for evaluation.

Dataset	Modality	Images	Categories
ChestXRay2017	X-ray	5856	Normal/Bacterial/Viral
BUSI	Ultrasound	780	Normal/Benign/Malignant
Brain Tumor MRI	MRI	7023	Glioma/Meningioma/Pituitary/Normal
Private Lymph Node	Ultrasound	1038	Benign/Malignant

**Table 2:** Quantitative comparison of methods on the BUSI dataset. All metrics are reported as percentages except AUC. The best results are highlighted in bold.

Method	Acc (%)	AUC	Sen (%)	Spe (%)
ResNet-50	85.99	0.9578	83.21	91.44
Swin-S	89.17	0.9713	89.39	93.95
Swin-B	89.81	0.9798	89.05	94.21
ViT-S/16	88.54	0.9654	88.22	93.82
ViT-B/16	89.81	0.9758	89.71	94.46
MedMamba-T	88.55	0.9528	89.92	94.78
MedMamba-B	89.06	0.9467	89.52	95.50
SVM	84.62	0.9410	69.05	92.05
XGBoost	87.69	0.9567	73.81	94.32
Random Forest	84.62	0.9175	69.05	92.05
**RadioGuide-DCN (ours)**	**93.63**	**0.9853**	**94.55**	**96.33**

Table 2 shows that our proposed RadioGuide-DCN achieves superior performance on the BUSI dataset compared to all baselines.

**Table 3:** Quantitative comparison on the Private Lymph Node Ultrasound dataset. All metrics are expressed as percentages except AUC.

Method	Acc (%)	AUC	Sen (%)	Spe (%)
ResNet-50	86.33	0.9150	83.81	87.23
Swin-S	88.91	0.9584	89.13	90.02
Swin-B	90.42	0.9675	90.27	91.54
ViT-S/16	88.07	0.9541	87.65	89.44
ViT-B/16	89.58	0.9621	89.90	90.62
MedMamba-T	89.96	0.9634	90.55	90.74
MedMamba-B	90.83	0.9688	91.02	91.56
SVM	89.37	0.9452	88.50	90.43
XGBoost	89.37	0.9666	92.92	85.11
Random Forest	87.44	0.9626	90.27	84.04
RadioGuide-DCN (ours)	92.75	0.9753	92.04	93.62

**Table 4:** Comparison of classification performance on ChestXRay2017 and Brain Tumor MRI datasets.The ChestXRay2017 baseline results are partially referenced from [42].

Method	ChestXRay2017		Brain Tumor MRI	
	Acc (%)	AUC	Acc(%)	AUC
ResNet-50	88.4	0.962	93.8	0.977
Swin-S	90.3	0.962	96.9	0.982
Swin-B	91.8	0.971	98.1	0.988
ViT-S/16	89.7	0.956	96.3	0.979
ViT-B/16	90.9	0.964	97.9	0.987
MedMamba-T	89.9	0.965	95.6	0.981
MedMamba-B	92.5	0.973	98.2	0.991
SVM	85.0	0.921	90.2	0.952
XGBoost	87.5	0.935	92.3	0.962
Random Forest	84.6	0.911	89.9	0.948
RadioGuide-DCN (ours)	93.4	0.984	98.3	0.993

**Table 5:** Ablation on BUSI. Accuracy (Acc), Sensitivity (Sen), and Specificity (Spe) are reported as percentages; AUC is in [0, 1].

Config	Acc (%)	AUC	Sen (%)	Spe (%)
Baseline	90.10	0.9750	89.45	94.56
+ACA	90.88	0.9765	93.50	95.30
+ACA+Deco	92.90	0.9839	94.00	95.80
+ACA+Deco+KAN (Full)	93.63	0.9853	94.55	96.33

## Data Availability

The original contributions presented in the study are included in the article; further inquiries can be directed to the corresponding author.
